# Effectiveness of Vildagliptin in Clinical Practice: Pooled Analysis of Three Korean Observational Studies (the VICTORY Study)

**DOI:** 10.1155/2017/5282343

**Published:** 2017-08-24

**Authors:** Sunghwan Suh, Sun Ok Song, Jae Hyeon Kim, Hyungjin Cho, Woo Je Lee, Byung-Wan Lee

**Affiliations:** ^1^Division of Endocrinology, Dong-A University Medical Center, Dong-A University School of Medicine, Busan, Republic of Korea; ^2^Division of Endocrinology, Department of Internal Medicine, National Health Insurance Service, Ilsan Hospital, Ilsan, Republic of Korea; ^3^Division of Endocrinology and Metabolism, Department of Medicine, Samsung Medical Center, Sungkyunkwan University School of Medicine, Seoul, Republic of Korea; ^4^Novartis Korea, Seoul, Republic of Korea; ^5^Department of Internal Medicine, Asan Medical Center, University of Ulsan College of Medicine, Seoul, Republic of Korea; ^6^Division of Endocrinology and Metabolism, Department of Internal Medicine, Severance Hospital, Yonsei University College of Medicine, Seoul, Republic of Korea

## Abstract

The present observational study aimed to evaluate the clinical effectiveness of vildagliptin with metformin in Korean patients with type 2 diabetes mellitus (T2DM). Data were pooled from the vildagliptin postmarketing survey (PMS), the vildagliptin/metformin fixed drug combination (DC) PMS, and a retrospective observational study of vildagliptin/metformin (fixed DC or free DC). The effectiveness endpoint was the proportion of patients who achieved a glycemic target (HbA1c) of ≤7.0% at 24 weeks. In total, 4303 patients were included in the analysis; of these, 2087 patients were eligible. The mean patient age was 56.99 ± 11.25 years. Overall, 58.94% patients achieved an HbA1c target of ≤7.0% at 24 weeks. The glycemic target achievement rate was significantly greater in patients with baseline HbA1c < 7.5% versus ≥7.5% (84.64% versus 43.97%), receiving care at the hospital versus clinic (67.95% versus 52.33%), and receiving vildagliptin/metformin fixed DC versus free DC (70.69% versus 55.42%). Multivariate logistic regression analysis indicated that disease duration (*P* < 0.0001), baseline HbA1c (*P* < 0.0001), and DC type (*P* = 0.0103) had significant effects on drug effectiveness. Vildagliptin plus metformin appeared as an effective treatment option for patients with T2DM in clinical practice settings in Korea.

## 1. Introduction

Type 2 diabetes mellitus (T2DM) is a well-established disease that causes disability (blindness, limb amputation, kidney failure, or cardiovascular events) in affected patients [1]. Since 1980, the age-adjusted prevalence of diabetes in adults has increased, which has resulted in quadrupling of the number of affected adults with diabetes in countries worldwide [2]. The burden of diabetes, both in terms of prevalence and number of adults affected, has rapidly increased in East Asian countries, including Korea [2, 3].

Among oral hypoglycemic agents (OHAs), dipeptidyl peptidase 4 (DPP-4) inhibitors are classified as a relatively new category which produce effects by increasing the concentration of active forms of incretin, such as glucagon-like peptide-1 (GLP-1) and glucose-dependent insulinotropic peptide (GIP). Thus, DPP-4 inhibitors can reduce fasting and postprandial blood glucose levels through effects on incretins by consequently increasing both *α*- and *β*-cell sensitivities to glucose levels [4]. The use of DPP-4 inhibitors in patients with T2DM has markedly increased in clinical practice because these are generally weight neutral and have a low risk of hypoglycemia [5]. The American Diabetes Association (ADA) and European Association for the Study of Diabetes (EASD) guidelines suggest the use of metformin as a first-line drug treatment and recommend the addition of a second drug if glycemic control is not achieved within the target levels [6]. In contrast to EASD/ADA, Japanese Diabetes Society (JDS) allows the use of any antidiabetic drugs that are appropriate to the pathophysiology of patient's diabetes [7]. Based on the guideline and potential, the incretin-based drug especially DPP-4 inhibitor is considered as a first choice therapy in Japanese type 2 diabetes patients [8]. Among DPP-4 inhibitors, vildagliptin is known to be an effective and safe therapeutic option for patients with T2DM, both as monotherapy and in combination with other medications [9]. Although the efficacy and safety of vildagliptin as monotherapy, dual therapy (particularly as an add-on to metformin), and triple therapy have been proven in randomized clinical trials (RCTs) [9], data regarding the effectiveness of vildagliptin in clinical practice settings, particularly in Korea, are scarce. Therefore, based on a pooled analysis of three studies conducted in clinical practice settings, we aimed to assess the glycemic effectiveness of vildagliptin plus metformin treatment in Korean patients with T2DM (the VICTORY study).

## 2. Methods

We pooled and analyzed data retrieved from prospective, phase 4, postmarketing surveillance (PMS) studies for vildagliptin and vildagliptin/metformin fixed drug combination (DC) as well as from a retrospective study of vildagliptin/metformin (fixed or free DC) (the VICTORY study). The primary endpoint of vildagliptin and vildagliptin/metformin fixed DC PMS studies was safety analysis. These were noninterventional “real-world” studies without any defined study-related procedures and were sponsored by Novartis Korea and conducted after the corresponding protocols were approved by the Ministry of Food and Drug Administration and institutional review board (IRB) (Severance Hospital, IRB number: 4-2010-0800). The retrospective observational study of vildagliptin/metformin, considered in the present analysis, was conducted after its protocol was approved by the IRB (Ulsan University Hospital, IRB number: 2012-075). The present study was conducted in accordance with the ethical principles stated in the Declaration of Helsinki and in compliance with the International Conference on Harmonization-Good Clinical Practice guidelines, as revised in 2013. Informed consent was obtained from all patients included in these studies. The study population comprised Korean individuals aged ≥ 19 years with T2DM, who were prescribed vildagliptin + metformin as combination therapy with or without other drug(s) in the form of add-on or initial combinations or vildagliptin/metformin as fixed DC. Extensive exclusion criteria were applied for this study. Details regarding inclusion and exclusion criteria of each of the studies are summarized in Supplementary Table S1 available online at https://doi.org/10.1155/2017/5282343.

The duration of the study was 24 weeks. At baseline, demographic data regarding gender, age, weight, treatment center type, concurrent disease, concomitant medication, medical history, duration of diabetes, and DC type were collected. In addition, laboratory data were collected at baseline, after an interim of 12 weeks, and at the final visit (approximately 24 weeks after the baseline visit). For effectiveness analysis, the achievement rate of the glycemic target (HbA1c ≤ 7.0%) at 24 weeks was assessed as the primary outcome. Secondary outcomes included changes in HbA1c levels and fasting plasma glucose levels at weeks 12 and 24. All endpoints are descriptively summarized at each visit. Continuous variables are expressed using descriptive statistics (*n*, mean ± SD), whereas discrete variables are summarized using frequency tables (*n*, %). We conducted chi-square tests and *t*-tests to evaluate differences between the groups. Multivariate regression analysis was used to determine correlations between the HbA1c target achievement rate and gender, age, weight, concurrent disease, medical history, concomitant medications, duration of disease, baseline HbA1c, and DC type. All data were analyzed using SAS (version 9.4, SAS Institute Inc., Cary, NC), and *P* values < 0.05 were considered statistically significant.

## 3. Results

**Figure 1:**
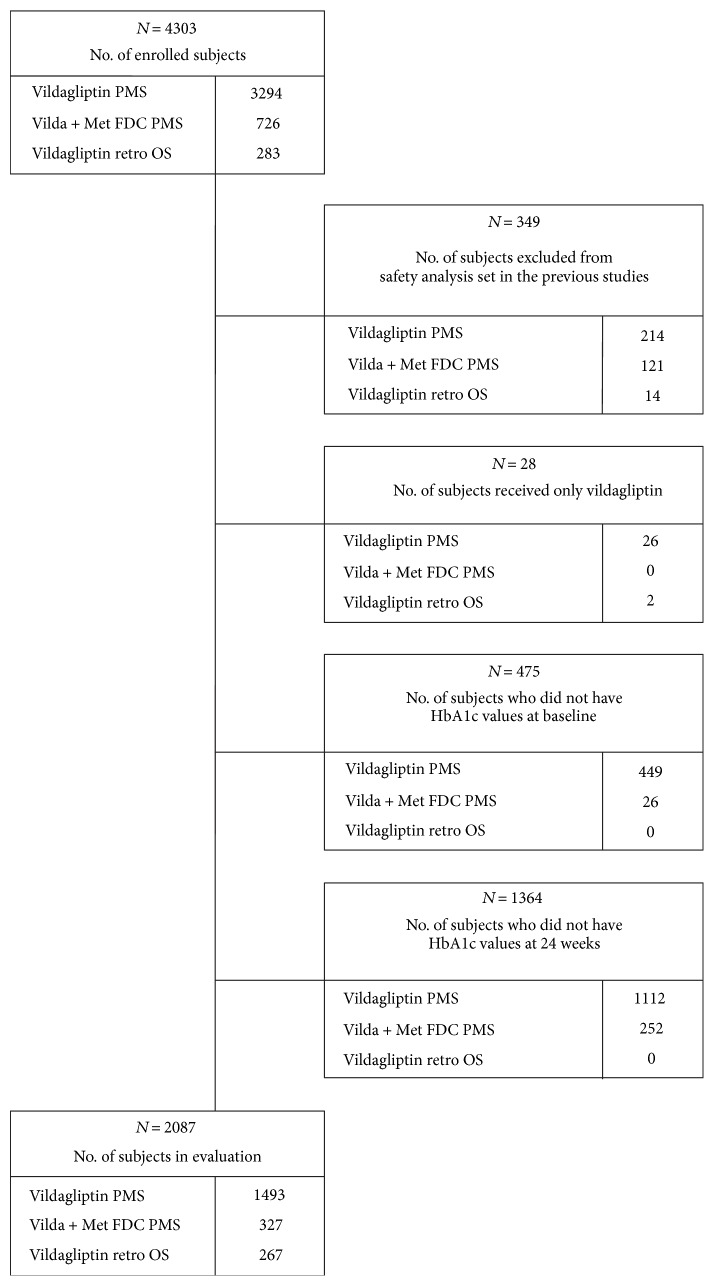
Flow diagrams of patient disposition. PMS: postmarketing survey; retro OS: retrospective observational study.

A total of 4303 patients who received treatment with vildagliptin at least once constituted the full analysis set in the three pooled studies (3294 from the vildagliptin PMS, 726 from the vildagliptin/metformin fixed DC PMS, and 283 from the vildagliptin retrospective study); of these, 2216 patients were excluded for the following reasons: violation of inclusion/exclusion criteria and/or the instructions regarding dosage and administration (*n* = 349), prescription of vildagliptin alone (*n* = 28), no documented baseline HbA1c (*n* = 475), and no documented HbA1c at 24 weeks (*n* = 1364) (Figure 1).

### 3.1. Baseline Characteristics according to Achievement of Target HbA1c Levels at 24 Weeks

Baseline characteristics of the study patients are presented in Table 1. The mean age and diabetes duration were 57 years and 6.24 years, respectively. Men accounted for 54.8% of the study population. Approximately 94% of patients were receiving dual therapy of vildagliptin and metformin. To identify the clinical factors that could affect the glycemic target achievement rate, the patients were divided into two groups according to achievement of the target HbA1c level of 7.0% at 24 weeks: good responder group I (HbA1c ≤ 7.0%; *n* = 1230; 695 men, 535 women) and nonresponder group II (HbA1c > 7.0%; *n* = 857; 449 men, 408 women). Overall, 58.9% of patients achieved the glycemic target (HbA1c ≤ 7.0%) at 24 weeks. No significant differences were noted in terms of gender or age between the groups. The duration of diabetes was significantly longer in group II (5.1 years in group I versus 7.7 years in group II). Moreover, the healthcare facility at which treatment was received also significantly differed between the groups (Table 1).

### 3.2. Glucose-Lowering Effectiveness according to Baseline Characteristics

**Figure 2:**
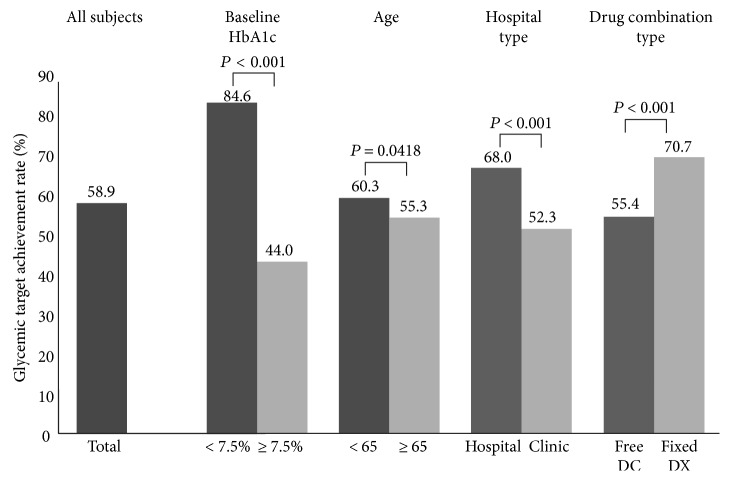
Proportion of patients achieving the glycemic target (HbA1c ≤ 7.0%) at 24 weeks. Free DC: free drug combination; Vilda/Met FDC: vildagliptin + metformin fixed dose combination.

In this analysis, the HbA1c levels decreased from 8.0 ± 1.4% at baseline to 7.0 ± 1.0% at the 24-week endpoint, which resulted in a significant reduction of 1.0 ± 1.3%. We also compared glucose-lowering effectiveness according to baseline characteristics. With regard to the DC type, the proportion of patients who achieved the target HbA1c was significantly greater in those treated with vildagliptin/metformin fixed DC compared with those treated with the free DC regimen (70.7% versus 55.4%). With regard to the healthcare facility at which treatment was received, the proportion of patients who achieved the glycemic target was greater in the hospital group than in the clinic group (68.0% versus 52.3%). Patients with baseline HbA1c < 7.5% showed a higher glycemic target achievement rate than patients with baseline HbA1c ≥ 7.5% (84.6% versus 44.0%). Overall, 60.3% of patients aged < 65 years achieved the glycemic target, whereas 55.3% of patients aged ≥ 65 years achieved the glycemic target at 24 weeks (Figure 2). The results of multivariate logistic regression models are presented in Table 2. After adjusting for other covariates, including gender, age, weight, concurrent disease, medical history, and concomitant medications, multivariate logistic regression analysis indicated that patients with lower baseline HbA1c and fixed DC type treatment exhibited 8.3- and 1.65-fold better outcomes, respectively, compared to those with higher baseline HbA1c (coefficient, 2.12; odds ratio, 8.3; 95% confidence interval (CI), 6.34–10.86) and free DC type treatment (coefficient, 0.5; odds ratio, 1.65; CI, 1.13–2.41); however, patients with a longer diabetes duration exhibited 0.92-fold poorer outcomes (coefficient, −0.08; odds ratio, 0.92; CI, 0.90–0.94) than did those with a shorter diabetes duration. None of the other variables exhibited a significant association with outcomes.

### 3.3. Adverse Events

Two patients reported hypoglycemia, and two patients reported elevated amylase or lipase from the PMS data. However, there was no report of heart failure or pancreatitis.

## 4. Discussion

Based on the analyses from three observational studies—including two 24-week PMS studies and one retrospective analysis of 50 mg twice daily vildagliptin in combination with metformin—in Korean patients with T2DM (VICTORY study), we demonstrated three primary findings. First, the use of vildagliptin as a second OHA significantly achieved reduction of HbA1c to the target level. Second, the clinical effectiveness of vildagliptin in combination with metformin in clinical practice settings is similar to that observed in RCTs. Third, dual therapy with vildagliptin and metformin as fixed DC induced better glycemic effects compared with free DC.

DPP-4 inhibitors have become commonly recommended drugs for glycemic control in patients with T2DM because these do not present the limitations exhibited by other OHAs [5]. Although the clinical relevance of effectiveness of DPP-4 inhibitors in terms of glycemic control remains unclear, such relevance is usually established based on data collected via RCTs. Nevertheless, the limitations of RCTs should be overcome by confirming these findings in real-world studies under clinical practice settings [10, 11]. In particular, RCTs narrowly define the inclusion and exclusion criteria to address the aims of a study, and hence, enrolled participants tend to be more highly motivated regarding their health status and care. Thus, such an analysis would provide only limited useful information regarding the effectiveness of a drug in real-world settings. In contrast, an observational study conducted in routine practice settings is more likely to enroll a broader patient population without any stringent inclusion/exclusion criteria and can thus obtain valuable information regarding the physician's preference and ideas as well as the real efficacy and side effects. However, at the expense of internal validity, real clinical practice is characterized by the lack of randomization, selection by the investigators, and the absence of a centralized laboratory and intensive monitoring. To address these limitations, there is an increasing call for pragmatic trials [10, 11]. Thus, the shortcomings of RCTs can be resolved through confirmation of findings via observational studies as part of routine care, which could help both patients and healthcare providers make better treatment decisions or improve adherence and outcomes. In the present study, we aimed to assess the clinical effectiveness of vildagliptin with metformin in clinical practice settings.

With regard to the glycemic effectiveness of vildagliptin—a potent and selective DPP-4 inhibitor—with metformin, we found that this combination was effective for HbA1c reduction, was weight neutral, and did not present any additional risk of hypoglycemia in RCTs [9, 12]. In a recent meta-analysis, add-on treatment with vildagliptin was found effective in reducing HbA1c (−0.67%), in comparison with placebo, in patients already treated with metformin. Similarly, patients treated with vildagliptin in addition to metformin exhibited no significant differences in the glycemic target achievement rates (HbA1c < 7%) in comparison with those treated with other active comparators [9]. Data from the VICTORY study are consistent with the key findings of these RCTs [9] and findings from previous real-life vildagliptin studies conducted in other regions [13–15]. The VICTORY study revealed that the mean reduction in HbA1c with vildagliptin as an add-on to metformin was −0.8% at the first follow-up visit at 12 weeks, which further improved to −1.0% at 24 weeks after treatment initiation. Interestingly, this HbA1c reduction with vildagliptin is better than previously reported results from RCTs (9). DPP-4 inhibitors are known to exhibit better glucose-lowering effects in Asians than in other ethnic groups. An earlier meta-analysis indicated that DPP-4 inhibitors lowered the HbA1c levels by 0.9% in studies with more Asian participants [16]. These findings might be explained by the varying contributions of insulin secretory defects and insulin resistance in the pathophysiological development of T2DM between Asians and non-Asians [17].

In the present study, patients with better baseline HbA1c and aged < 65 years exhibited better glycemic target achievement at 24 weeks; this could be explained by the shorter duration of diabetes, better self-management, and remaining *β*-cell function in this group [18, 19]. In addition, a larger proportion of patients treated at hospitals achieved the glycemic target compared with those treated at clinics; this may be owing to the comprehensive management by an endocrinology team (including an endocrinologist, nurse, and dietitian) in hospital settings, which was also cited in another Korean study [20].

Despite the progress in our understanding of the pathogenesis of diabetes and the development of new drugs, the present levels of care for patients with T2DM remain unsatisfactory [21]. Considering the complexity and progressive nature of T2DM, monotherapy might not yield long-term benefits. Therefore, even at the time diabetes is diagnosed, it might be appropriate to consider combination therapies to achieve adequate glycemic control in patients with T2DM [19]. In addition, the Global Partnership for Effective Diabetes Management recommends a more proactive approach and advocates earlier use of combination therapy in parallel with diet and exercise reinforcement for the management of T2DM [22]. Moreover, combination therapy in T2DM should address the various pathophysiological mechanisms that cause hyperglycemia [23]. Metformin primarily ameliorates insulin resistance, whereas DPP-4 inhibitors improve pancreatic islet cell function by maintaining the bioactivity of endogenous GLP-1. Therefore, coadministration of these two classes of antidiabetic medications could yield synergistic effects in the management of T2DM [24]. In fact, combination treatment with metformin and DPP-4 inhibitors induced a greater reduction in HbA1c levels compared with monotherapy alone. In addition, combination treatment showed good safety profiles, including a low risk of hypoglycemia and weight neutrality [24]. Furthermore, a meta-analysis of the initial combination of DPP-4 inhibitors with metformin showed potential benefits of this therapy on glycemic outcomes, compared with metformin monotherapy alone, across a wide range of baseline HbA1c levels [25]. Metformin was also shown to enhance secretion of GLP-1, which possibly improved the effectiveness of DPP-4 inhibitors [26].

Moreover, drug adherence is a critical factor to consider in the management of T2DM. The discontinuation of antidiabetic medication leads to a significant cost burden on the healthcare system and is frequently encountered in primary care patients [15, 27]. Furthermore, patients with T2DM often have metabolic comorbid conditions such as hypertension and dyslipidemia [28]. Management of such metabolic comorbidities increases the pill burden on patients, which could lead to an increased risk of medication-related problems, such as drug-drug interactions and adverse events, as well as poor treatment adherence [19, 27]. Hence, single-pill fixed DC medication may help patients with diabetes to achieve their glycemic targets and promote adherence through reduced pill and cost burdens [19]. Thus, fixed DC can enhance drug adherence and should be considered in patients with chronic diseases such as T2DM to improve drug adherence, which may consequently lead to better clinical outcomes [27, 29, 30]. With regard to the clinical relevance of fixed DC therapy, in the present study, vildagliptin plus metformin dual therapy as fixed DC exhibited better glycemic effectiveness in our subgroup analysis and multivariate analysis (Figure 2 and Table 2).

The strengths of the VICTORY study include the large sample examined under clinical practice settings, which can provide valuable additional information on vildagliptin. However, the study also had certain limitations. First, the study did not investigate the mechanism of action of vildagliptin and did not seek to alter the treatment guidelines. Second, this study had a nonrandomized, open-label, uncontrolled design, which may be associated with potential observer and selection bias. The final limitation was the open nature of the trial, which allowed doctors to select any drug for treatment, although this was expected given that the study reflects actual clinical practice settings.

## 5. Conclusion

The results of this study conducted in real-world clinical practice settings in Korea demonstrate that vildagliptin plus metformin is a clinically reasonable option as a combination therapy for patients with T2DM.

## Supplementary Material

Supplementary Table 1. Inclusion and exclusion criteria of enrolled study.

## Figures and Tables

**Table 1:** Baseline patient characteristics according to HbA1c levels at 24 weeks.

	HbA1c ≤ 7.0% at 24 weeks (*N* = 1230)	HbA1c > 7.0% at 24 weeks (*N* = 857)	Total (*N* = 2087)	*P* value
Gender, *n* (%)				
Male	695 (56.5)	449 (52.4)	1144 (54.8)	0.0633^a^
Age (years)				
Mean ± SD	56.8 ± 11.0	57.3 ± 11.7	57.0 ± 11.3	0.2517^b^
Weight (kg)				
*N*	1092	778	1870	
Mean ± SD	67.5 ± 11.0	66.3 ± 10.9	67 ± 11.0	0.0215^b^
Baseline HbA1c, *n* (%)				
<7.5%	650 (52.9)	118 (13.8)	768 (36.8)	<0.0001^a^
≥7.5%	580 (47.2)	739 (86.2)	1319 (63.2)	
Elderly group, *n* (%)				
<65 years	926 (75.3)	611 (71.3)	1537 (73.7)	0.0418^b^
≥65 years	304 (24.7)	246 (28.7)	550 (26.4)	
Treatment center type, *n* (%)				
Hospital	600 (48.8)	283 (33.0)	883 (42.3)	<0.0001^b^
Clinic	630 (51.2)	574 (67.0)	1204 (57.7)	
Concurrent disease, *n* (%)				
Yes	700 (56.9)	449 (52.4)	1149 (55.1)	0.0412^b^
No	530 (43.1)	408 (47.6)	938 (44.9)	
Medical history, *n* (%)				
Yes	153 (12.4)	83 (9.7)	236 (11.3)	0.0296^b^
No	1036 (84.2)	756 (88.21)	1792 (85.9)	
Concomitant medications (except for diabetes medications), *n* (%)				
Yes	733 (59.6)	448 (52.3)	1181 (56.6)	0.0009^b^
No	497 (40.4)	409 (47.7)	906 (43.4)	
Duration of T2DM (years)				
*n*	1067	785	1852	
Mean ± SD	5.1 ± 5.3	7.7 ± 6.0	6.2 ± 5.8	<0.0001^a^
Drug combination type, *n* (%)				
Vildagliptin + metformin^c^	895 (72.8)	720 (84.0)	1615 (77.4)	<0.0001^b^
Vildagliptin/metformin FDC^d^	299 (24.3)	124 (14.5)	423 (20.3)	
Pharmacotherapy at baseline, *n* (%)				
Second-line therapy	1181 (96.0)	775 (90.4)	1956 (93.7)	<0.0001^b^
Third- or further-line therapy	43 (3.5)	76 (8.9)	119 (5.7)	
∆HbA1c (%)				
12 weeks				
*N*	923	699	1622	
Mean ± SD	−0.9 ± 1.1	−0.6 ± 1.2	−0.8 ± 1.2	<0.0001^b^
24 weeks				
*N*	1230	857	2087	
Mean ± SD	−1.2 ± 1.2	−0.8 ± 1.3	−1.0 ± 1.3	<0.0001^b^
∆FBG (mg/dL)				
12 weeks				
*N*	535	359	894	
Mean ± SD	−30.9 ± 47.1	−24.2 ± 57.7	−28.2 ± 51.7	0.0673^b^
24 weeks				
*N*	663	406	1069	
Mean ± SD	−32.6 ± 48.8	−23.0 ± 60.2	−29.0 ± 53.6	0.0068^b^

SD: standard deviation; T2DM: type 2 diabetes mellitus; FDC: fixed drug combination; FBG: fasting blood glucose. ^a^Chi-square test. ^b^*t*-test. ^c^Vildagliptin + metformin free drug combination. ^d^Vildagliptin/metformin fixed dose combination.

**Table 2:** Multivariate analysis for factors associated with glycemic target achievement (HbA1c < 7.0%) following vildagliptin treatment.

Variable	Coefficient	SE of coefficient	*Z* value	*P* value	Odds ratio	95% CI
Gender	0.06	0.14	0.21	0.645	1.07	0.81–1.41
Age	0.01	0.01	2.84	0.092	1.01	1.00-1.02
Weight	0.00	0.01	0.15	0.697	1.00	0.99–1.02
Concurrent disease	−0.25	0.23	1.13	0.288	0.78	0.49–1.24
Medical history	0.07	0.20	0.11	0.736	1.07	0.72–1.59
Concomitant medications (except for diabetes medications)	0.36	0.24	2.29	0.131	1.43	0.90–2.28
Duration of diabetes (1 year)	−0.08	0.01	52.72	<0.001	0.92	0.90–0.94
HbA1c < 7.5%	2.12	0.14	237.15	<0.001	8.30	6.34–10.86
Fixed dose combination treatment	0.50	0.19	6.58	0.010	1.65	1.13–2.41

SE: standard error; CI: confidence interval. Odds ratio of vidagliptin/metformin fixed dose combination was calculated in comparison with the free drug combination.
