# Supplementary material for: Effectiveness of Vildagliptin in Clinical Practice: Pooled Analysis of Three Korean Observational Studies (the VICTORY Study)
Source: J Diabetes Res. 2017 Aug 24;2017:5282343. doi: 10.1155/2017/5282343 (PMC5613692; doi:10.1155/2017/5282343)
Supplement: Supplementary file 1 — Supplementary Table 1. Inclusion and exclusion criteria of enrolled study. [file 5282343.f1.doc]

**Supplementary Table 1. Inclusion and exclusion criteria of enrolled study**

| Study | Inclusion criteria | Exclusion criteria |
| --- | --- | --- |
| Vildagliptin PMS | 1.Patients with type 2 diabetes mellitus aged 19 years or older | 1.Subjects with hypersensitivity to vildagliptin, an active ingredients of the study drug or any other ingredients of the study drug |
|  | 2.Subjects who can receive a monotherapy along with diet and exercise therapies, or | 2.Subjects with type 1 diabetes mellitus or with diabetic ketoacidosis |
|  | 3.Subjects who have not received diabetes treatment and can receive a combination with metformin if blood glucose cannot be controlled sufficiently with a monotherapy, or | 3.Women who are pregnant or breastfeeding |
|  | 4.Subjects whose blood glucose cannot be controlled sufficiently with a monotherapy of sulfonylurea, metformin or thiazolidinedione, or | 4.As the study drug contains lactose, patients with rare hereditary problems of galactose intolerance, the Lapp lactase deficiency or glucose–galactose malabsorption should not take this medicine |
|  | 5.Subjects whose blood glucose cannot be controlled sufficiently with a combination therapy of sulfonylurea and metformin |  |
|  | 6.Subjects whose blood glucose cannot be controlled sufficiently with insulin therapy (monotherapy of insulin or combination therapy of insulin and metformin) |  |
|  | 7.Subjects who are given clear explanation of the objectives and characteristics of the survey by the investigator, sign the informed consent form and participate in the study |  |
| Vildagliptin/metformin FDC PMS | 1.Subjects aged 19 years or older | 1.Patients with renal disease or renal dysfunction (serum creatinine levels ≥1.5 mg/dL in males and ≥1.4 mg/dL in females or abnormal creatinine clearance (<60 mL/ml)) which may also result from conditions such as cardiovascular collapse (shock), acute myocardial infarction, and septicemia |
|  | 2.Subjects of vildagliptin/metformin 50/500 mg: | 2.Patients with congestive heart failure requiring pharmacologic treatment |
|  | Subjects with insulin-independent diabetes mellitus (type 2) who receive diet and exercise therapies, do not have experience of diabetes drug treatment and cannot control blood glucose sufficiently with a monotherapy or who switch from combination of vildagliptin and metformin hydrochloride | 3.Patients undergoing radiologic procedures (e.g.: intravenous urography, intravenous cholangiography, angiography, computed tomography using contrast materials, etc.) involving intravascular administration of iodinated contrast materials (because use of such products may result in acute alteration of renal function and increase the risk of lactic acidosis. In patients undergoing such tests, vildagliptin/metformin should be temporarily discontinued at the time of or prior to the procedure, withheld for 48 hours subsequent to the procedure and reinstituted only after renal function has been re-evaluated and found to be normal) |
|  | 3.Subjects of vildagliptin/metformin 50/850 mg, 50/1000 mg: | 4.Patients with known hypersensitivity to vildagliptin/metformin or to any of the excipients |
|  | Subjects with insulin-independent diabetes mellitus (type 2) who receive diet and exercise therapies, do not have experience of diabetes drug treatment and cannot control blood glucose sufficiently with a monotherapy; who cannot control blood glucose sufficiently with a metformin hydrochloride monotherapy; who switch from combination of vildagliptin and metformin hydrochloride; who cannot control blood glucose sufficiently with combination of metformin and sulfonylurea and are given concomitant sulfonylurea; or who cannot control blood glucose sufficiently with combination of insulin and metformin and are given concomitant insulin. | 5. Patients with type 1 diabetes mellitus patients with acute or chronic metabolic acidosis, including diabetic ketoacidosis, with or without coma and patients with a history of ketoacidosis [Type1 diabetes mellitus and diabetic ketoacidosis should be treated with insulin]. |
|  | 4.Subjects who are given clear explanation of the objectives and characteristics of the survey by the investigator, sign the informed consent form and participate in the study | 6. Use of vildagliptin should be temporarily suspended in patients with severe infection or severe traumatic systemic disorder and should not be restarted until the patient’s oral intake has resumed and renal function has been evaluated as normal. Use of vildagliptin/metformin should be temporarily suspended for any surgical procedure (except minor procedures not associated with restricted intake of food and fluids) prior to 48 hours of the procedure and should not be restarted until the patient’s oral intake has resumed and renal function has been evaluated as normal after at least 48 hours. |
|  |  | 7.Patients with malnutrition, starvation, weakness, pituitary dysfunction or adrenal dysfunction |
|  |  | 8.Patients with hepatic impairment (since impaired hepatic function has been associated with some cases of lactic acidosis, vildagliptin/metformin should generally be avoided in patients with clinical or laboratory evidence of hepatic disease), pulmonary infarction, severe pulmonary dysfunction and any conditions associated with hypoxia, excessive alcohol intake, dehydration, gastro-intestinal impairment such as diarrhea, vomiting, etc. |
|  |  | 9.Women who are pregnant, possibly pregnant or breastfeeding |
| Vildagliptin, vildagliptin/metfomin retrospective study | 1.T2DM patients who have been given vildagliptin+metformin or vildagliptin/metformin FDC monotherapy for over 6 months | 1.Patients who do not have indications for vildagliptin+metformin or vildagliptin/metformin |
|  | 2. Patients with at least one HbA1c result before and 6 months after the first dose of vildagliptin+metformin or vildagliptin/metformin.FDC | 2.Patients given vildagliptin in combination with diabetes medications (including insulin) other than metformin |

PMS, post marketing surveillance; FDC, fixed drug combination
